# ‘Mini-Max’ knotless acetabular labrum repair: repair construct rationale and allocation in a consecutive case series with minimum 1-year clinical outcomes

**DOI:** 10.1093/jhps/hnab061

**Published:** 2021-08-30

**Authors:** John J Christoforetti, Gabriella Bucci, Beth Nickel, Steven B Singleton, Ryan P McGovern

**Affiliations:** Department of Orthopedic Sports Medicine and Hip Preservation Surgery, Texas Health Orthopedic Specialist, Dallas/ Ft Worth, 5858 Main St. Suite 210, Frisco, TX 75034, USA; Department of Orthopedic Sports Medicine and Hip Preservation Surgery, Allegheny Singer Research Institute, 4800 Friendship Ave, Pittsburgh, PA 15224, USA; Department of Orthopedic Sports Medicine and Hip Preservation Surgery, Texas Health Orthopedic Specialist, Dallas/ Ft Worth, 5858 Main St. Suite 210, Frisco, TX 75034, USA; Department of Orthopedic Sports Medicine and Hip Preservation Surgery, Allegheny Singer Research Institute, 4800 Friendship Ave, Pittsburgh, PA 15224, USA; Department of Orthopedic Sports Medicine and Hip Preservation Surgery, Texas Health Orthopedic Specialist, Dallas/ Ft Worth, 5858 Main St. Suite 210, Frisco, TX 75034, USA; Department of Orthopedic Sports Medicine and Hip Preservation Surgery, Texas Health Orthopedic Specialist, Dallas/ Ft Worth, 5858 Main St. Suite 210, Frisco, TX 75034, USA; Department of Orthopedic Sports Medicine and Hip Preservation Surgery, Allegheny Singer Research Institute, 4800 Friendship Ave, Pittsburgh, PA 15224, USA

## Abstract

To describe the ‘mini-Max’ approach to labrum repair using non-absorbable 2.4-mm knotless suture anchors and report objective clinical outcomes with a large single-surgeon cohort. Level 3 retrospective case series. A retrospective review was conducted to report the use and allocation of non-absorbable 2.4-mm knotless suture anchors during ‘mini-Max’ labral repair from 2015 to 2018. Descriptive analysis of the labral damage severity, size and number of anchors used to arthroscopically repair the acetabular labrum was performed. Paired-samples t-tests were performed to evaluate whether preoperative and 1-year follow-up patient-reported outcomes (PROs) were statistically significant. An analysis of variance was performed comparing PROs with categorized number of labral anchors. A total of 390 patients were queried in this study, with 330 (85%) diagnosed intraoperatively with acetabular labral tears. A total of 245 patients (137 females and 108 males) with a mean age of 30.1 ± 11.6 years (mean ± SD) at the time of surgery underwent ‘mini-Max’ labral refixation. Of the 245 labral tears, 88 (35.9%) were graded as mild, 113 (46.1%) as moderate and 44 (18.0%) as severe. Labral repairs required an average of 2.1 ± 0.67 anchors across all patients included. Forty-one repairs (16.7%) required one anchor, 139 (56.7%) required two anchors, 63 (25.7%) required three anchors and 2 (0.8%) required four anchors. Significant improvements were reported for all PROs (*P* ≤ .001) at a minimum of 1-year follow-up. Arthroscopic ‘mini-Max’ labral repair using non-absorbable knotless suture anchors is a safe and effective technique for improving the lives of patients suffering from symptomatic acetabular labrum tears.

## INTRODUCTION

Over the past decade, there has been a shift in the arthroscopic treatment of hip labral pathologies from predominantly debridement to an increase in labral repair [1, 2]. A recent systematic review reported that between 2009 and 2017 there was an increase of labral repairs from 19% to 81% of cases, respectively [1]. In 2015, data collected from the American Board of Orthopaedic Surgery Database showed that 79.2% of hip arthroscopy cases reported by candidates included labral repair [2]. This could be attributed to a growing number of studies that demonstrate superior clinical outcomes and a lower risk of conversion to arthroplasty associated with labral repair when compared to debridement or partial resection of the labrum [1, 3].

Arthroscopic labral repair is a highly specialized procedure with a challenging learning curve [2, 4]. A recently clarified surgical principle includes preservation of the chondrolabral junction and reconstitution of the labrum to efficiently preserve blood flow and increase the likelihood of healing [5–7]. The surgical technique and properties of the anchors used for refixation play a significant role in obtaining successful results [4, 7–10]. Precise placement of sutures, anchors and careful re-tensioning of the labrum are imperative for restoring the suction seal [1, 4, 7]. Various labral repair techniques have been described, including simple loop, cinch, modified cinch and a labral base repair technique without clear superiority of one technique [4, 8, 11]. A wide selection of sutures, anchors and materials from different medical manufacturers allow orthopedic surgeons to select the appropriate equipment based on their personal preferences and abilities [4, 8–10].

The ‘mini-Max’ technique of fracture fixation described by Weber balances the disruption to the native soft tissues and the use of minimally required hardware to achieve maximal repair construct efficacy [12]. The principles established for this technique can be applied to the preparation and repair of the labrum during hip arthroscopy. For the ‘mini-Max’ labral repair, a similar approach is taken whereby minimal capsulolabral tissue is disrupted that is required for bone preparation and repair and utilization of the fewest possible suture anchors for stable tissue approximation and healing. The purpose of the current study is to describe the ‘mini-Max’ approach to labrum repair using non-absorbable 2.4-mm knotless suture anchors and report objective clinical outcomes with a large single-surgeon cohort. The ‘mini-Max’ philosophy of soft-tissue management and repair construct selection is also described.

## METHODS

### Patient selection

A retrospective review was conducted to report the allocation of non-absorbable 2.4-mm knotless suture anchors during ‘mini-Max’ labral repair and the effect of these suture anchors on clinical outcomes at a minimum of 1-year follow-up. From July 2015 to October 2018, data were prospectively collected on 390 patients undergoing primary hip arthroscopy for intra-articular pathology by the first author (JJC). All subjects and parents/guardians (when applicable) approved and signed the written informed consent and authorization to disclose protected health information for a research study established under the Allegheny Singer Research Institute institutional review board. Inclusion criteria for this study included patients who were able to consent for participation, parental/guardian permission (informed consent) and if appropriate; child assent, the ability to read and understand English and consent for themselves; age 14–60 years; intraoperatively repaired acetabular labral tear in isolation and/or with one or more of the following pathological findings: acetabular chondrosis, femoral head chondrosis, cam and/or pincer deformity femoroacetabular impingement syndrome, partial ligamentum teres tears and synovitis; and complete office medical records and operative note for specifics of acetabular labral repair during primary hip arthroscopy by the treating orthopedic surgeon. Exclusion criteria for this study included any patient failing to sign the informed consent, previous ipsilateral hip surgery, findings of dysplasia (<20° of acetabular coverage measure via radiographs and magnetic resonance imaging [MRI]) and evidence of advanced osteoarthritis (Tönnis 3).

### Preoperative clinical evaluation

Patient demographics that previously correlated with the impact on outcomes following pre-arthritic hip arthroscopy were recorded along with key physical examination findings including radiographic parameters and MRI results [13–15]. Patients were imaged with a weight-bearing superior anteroposterior (AP) view of the pelvis, a lateral view of the proximal femur (Dunn 45° view) and a standing false profile view of the pelvis [14, 16]. Preoperative radiographic measurements were made by a trained member of the research team blinded to the surgical method chosen. Anterior center edge angles, lateral center edge angles and alpha angles were recorded for all patients. Tönnis classification for osteoarthritis was assessed on the AP view and gives an objective evaluation for the severity of degeneration [17]. MRI techniques included imaging in the oblique plane along the femoral neck as well as standard coronal, sagittal and axial plane views of the hip and pelvis to evaluate for soft-tissue conditions of the hip joint and surrounding musculoskeletal structures [15].

Following physical examination and imaging, a diagnostic intra-articular injection was performed for all patients under ultrasound guidance by the senior author (JJC). After 5 min, the patient was re-evaluated by physical provocation maneuvers that were evaluated as painful prior to the diagnostic injection. The patient was then asked to rate their improvement on a scale of 0–100%. A positive injection response was reported if the patient’s symptoms improved by a minimum of 80% after injection. If no immediate improvement was reported by the patient, the injection was considered non-diagnostic. Prior to surgical consideration, all patients with a positive injection response performed a 6- to 8-week rehabilitation intervention focused on patient education, activity modification, limitation of aggravating factors, an individualized physical therapy program and a home-exercise program. Supervised physical therapy was provided by the rehabilitation specialist of the patients choosing 1 day a week. The home-exercise program distributed to the patients was from a previously performed literature review [18]. Participants completed four exercises of the home-exercise program on the weekdays when they were not participating in the individualized physical therapy intervention. The patients were instructed to cycle through the 12 total exercises during the week, while not repeating an individual exercise on back-to-back days. Patients with a positive diagnostic injection who failed conservative management and were diagnosed with chondrolabral pathologies by the treating orthopedic surgeon were recommended for primary hip arthroscopy.

### Intraoperative technique: ‘mini-Max’ knotless labrum repair

Patients were placed in a supine position on a hip arthroscopy minimal-post table after properly protecting the pressure areas. Traction was applied to the operative hip using a limb spar and fluoroscopic visualization. The hip was accessed via an anterolateral portal (ALP) with a 70-degree lens arthroscope. Subsequently, the mid-anterior portal was created, and an arthroscopic blade was used to perform either an interportal or periportal capsulotomy [19].

Routine diagnostic arthroscopy was performed with the assessment of central and peripheral compartments, including cartilage surfaces of the acetabulum and femoral head, ligamentum teres and labrum. Intraoperative details were recorded by the treating surgeon including operative procedures and standardized description of diagnostic arthroscopic findings. Upon identification of acetabular labral tears, each was graded for damage as mild, moderate or severe based upon the following criteria: mild—no disruption of labrum base or capsulolabral tissue, minimal intrasubstance damage; moderate—disruption of capsulolabral or labrum base tissue, minimal intrasubstance damage; severe—disruption of labral base and capsulolabral integrity, severe intrasubstance damage. When acetabular labral repair was determined as the appropriate procedure by the treating orthopedic surgeon, the number of anchors placed and the extent of anteromedial and posterolateral labral injury using the clock-face method for each patient were recorded [20]. As a standard, the 3 o’clock position was used to denote the anterior extent and the 9 o’clock position the posterior extent, regardless of sidedness (left or right).

**Fig. 1.**
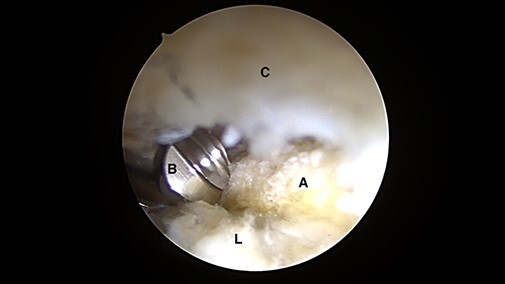
Intra-articular arthroscopic views from ALP representing the ‘mini-Max’ labrum repair technique. Minimal invasive acetabuloplasty is performed using a manual rasp under direct visualization preserving the chondrolabral junction without detaching the labrum (C = capsule; A = acetabulum; L = labrum; B = manual rasp).

**Fig. 2.**
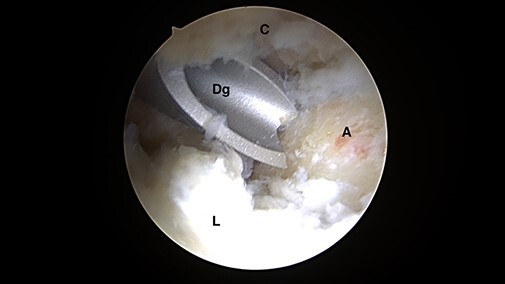
Intra-articular arthroscopic views from ALP representing the ‘mini-Max’ labrum repair technique. A straight drill guide is positioned on the prepared acetabulum rim, avoiding penetration to both the articular surface and the deep psoas canal (C = capsule; A = acetabulum; L = labrum; Dg = drill guide).

After identifying the area of labrum damage, the ‘mini-Max’ technique calls for the preservation of all intact chondrolabral junctional and capsulolabral junctional tissues. This is accomplished by the use of an arthroscopic elevator to ‘peel’ the capsulolabral to chondrolabral complex from the underlying rim bone, without transection of this continuous tissue sleeve. The management of the acetabular rim is determined by the integrity of the chondrolabral juncture. If the chondrolabral juncture remains intact, a minimal invasive acetabuloplasty is performed using a manual rasp without detaching the labrum if no formal acetabuloplasty is required for correction of retroversion or overcoverage (Fig. 1). An additional distal anterolateral portal may be created if needed for a better angle of approach. A straight drill guide is positioned on the prepared acetabulum rim, avoiding penetration to both the articular surface and the deep psoas canal (Fig. 2). Visualization of the articular surface was maintained throughout the course of drilling. A guidewire was then used to sound the pilot drill hole to verify an intact deep tunnel wall.

**Fig. 3.**
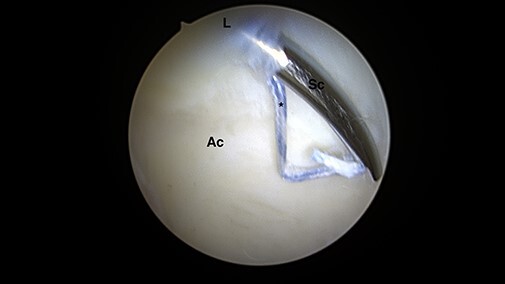
Intra-articular arthroscopic views from ALP representing the ‘mini-Max’ labrum repair technique. Placement of the suture between the rim of the acetabulum and the labral base tissue using a small anterograde suture passer (NanoPass, Stryker, USA). (L = labrum; Ac = acetabular cartilage; Sc = suture passer device; *suture).

**Fig. 4.**
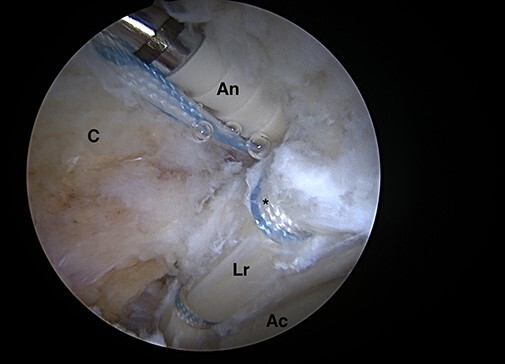
Intra-articular arthroscopic views from ALP representing the ‘mini-Max’ labrum repair technique. The torn labrum is secured using a non-absorbable PEEK 2.4-mm knotless PushLock® (Arthrex, Inc., Naples, FL, USA) to complete the knotless repair by seating the anchor into the pilot drill hole with a low-profile repair construct with a simple suture configuration (Lr = labrum repaired; C = capsule; Ac = acetabular cartilage; An = anchor; *suture).

**Fig. 5.**
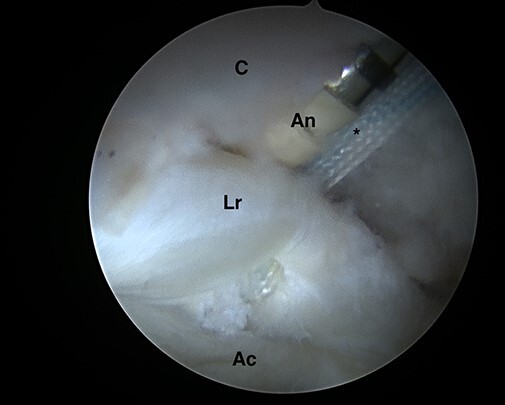
Intra-articular arthroscopic views from the ALP representing the ‘mini-Max’ labrum repair technique. The torn labrum is secured using a non-absorbable PEEK 2.4-mm knotless PushLock® (Arthrex, Inc., Naples, FL, USA) to complete the knotless repair by seating the anchor into the pilot drill hole with a low-profile repair construct with a labral base suture configuration (Lr = labrum repaired; C = capsule; Ac = acetabular cartilage; An = anchor; *suture).

**Fig. 6.**
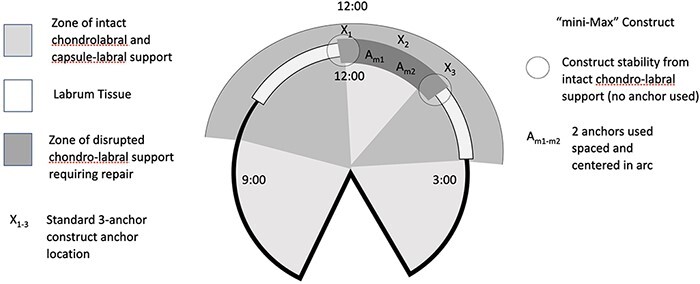
Illustrative diagram representing the ‘mini-Max’ construct with two anchors spaced and centered in the arc or labrum tissue tear (A_m1_, A_m2_) versus a standard construct using three anchors (X_1_, X_2_, X_3_). A distance of 5–10 mm is left between each anchor and evenly centered within the arc of repair.

**Fig. 7.**
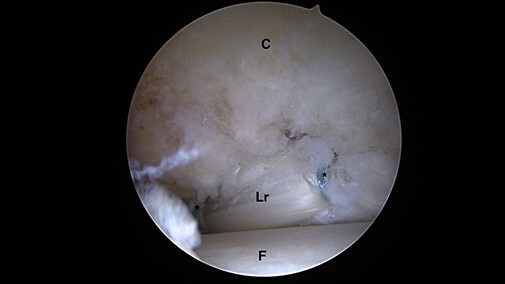
Intra-articular arthroscopic view of the right hip, with traction off, representing the free edge of the repaired labrum and the femoral head. The suction-seal function of the repaired labrum is observed (Lr = labrum repaired; C = capsule; F = femoral head; *suture).

The suture was placed between the rim of the acetabulum and the labral base tissue. (Fig. 3) The suture was released making certain that it was not incarcerated and that it was positioned radially across from the drill hole. The torn labrum was then secured using a small anterograde suture passer (NanoPass, Stryker, USA) to create a low-profile repair construct with either a simple or a mattress suture configuration (Figs 4 and 5, respectively). Suture pattern was either labral base or simple as described by Jackson *et al*. [21]. A simple stitch structure is usually preferred to repair small labrums (<3 mm), in which a labrum base stitch is not achievable or everts the labrum, loosening the suction-seal function. The sutures were captured and withdrawn through the ultimate suture placement portal and then secured using the non-absorbable PEEK 2.4-mm knotless PushLock® (Arthrex, Inc., Naples, FL, USA) to complete the knotless repair by seating the anchor into the pilot drill hole. A distance of 5–10 mm was left between each anchor and evenly centered within the arc of repair (Fig. 6). Traction was then released, and the suction-seal function of the hip was observed (Fig. 7). Associated procedures were performed concomitantly according to the patient’s diagnosis. Capsular closure was performed for all interportal capsulotomies. 

### Postoperative care

All patients received a standard postoperative regimen involving a continuum of physician and physical therapist–directed care. A detailed description is outlined in Appendix A.

### Patient-reported outcomes

Specific patient-reported outcomes (PROs) included the Hip Outcome Score—Activities of Daily Living (HOS-ADL) [22], Hip Outcome Score—Sports Specific Subscale (HOS-Sport) [23], the 12-item International Hip Outcome Tool (iHOT) [24] and visual analog scale (VAS) [25] for hip pain (0, no pain; 100, worst imaginable pain) were collected by a clinical outcomes expert (RPM) preoperatively and at a minimum of 1 year following surgical intervention. Patient satisfaction (0, not satisfied at all; 100, completely satisfied) was collected for each patient at a minimum of 1-year follow-up from surgical intervention. PROs were collected with a cloud-based software tracking system (OBERD© 2019 Universal Research Solutions, LLC, Columbia, MO, USA).

### Statistical analysis

Descriptive analysis of the labral damage severity, size (clock-face description) and number of anchors used to arthroscopically repair the acetabular labrum was performed. Fisher’s exact tests were performed to evaluate if other intraoperative procedures performed with labral repair in the 1-year follow-up group were a statistically significant representation of the entire population of this study. Paired-samples t-tests were performed to evaluate whether preoperative and 1-year follow-up PROs were statistically significant for all included patients. An analysis of variance was performed comparing PROs with categorized number of labral anchors. All statistical analyses were performed with an *a*  *priori* alpha set of P < 0.05. All data were analyzed using a common statistical software program (IBM SPSS Statistics, Version 25, Armonk, NY, USA).

## RESULTS

### Patient results

Of the 390 patients queried in this study, 330 (85%) were diagnosed intraoperatively with acetabular labral tears. After administering the inclusion criteria, 245 patients (137 female and 108 male) with a mean age of 30.1 ± 11.6 years (mean ± SD) at the time of surgery and body mass index (BMI) of 25.7 ± 4.6 underwent labral repair and were eligible to be included in the study. Intraoperative procedures performed concomitantly with labral repair are presented in Table I.

**Table I.** Intraoperative procedures performed with labral repair

	*Total* n*/245(%)*	*1-year follow-up* n*/162(%)*	*Significance* P*-value*
Acetabular chondroplasty	72 (29)	46 (28)	0.911
Acetabular microfracture	22 (9)	16 (10)	0.862
Acetabuloplasty	94 (38)	56 (35)	0.464
Femoral chondroplasty	15 (6)	9 (6)	1
Femoroplasty	124 (51)	87 (54)	0.545
Ligamentum teres debridement	49 (20)	33 (20)	1
Synovectomy	139 (57)	96 (59)	0.682

Of the 245 patients qualified for the 1-year follow-up, 162 (66%) patients (89 female and 73 male) had a mean age of 30.2 ± 11.7 years (mean ± SD) and a mean BMI of 25.7 ± 4.6 at the time of surgery. Intraoperative procedures performed with labral repair are also presented for this group of patients in Table I.

### Severity and size of labral tears

Of the 245 labral tears included in this study, 88 (35.9%) were graded as mild, 113 (46.1%) as moderate and 44 (18.0%) as severe. The circumferential size of the labral tears included in this study, as described by the number of ‘hours’, is presented in Table II. The most common size of tear was a ‘3-hour’ tear, accounting for 106 patients (43.3%) that underwent labral repair.

**Table II.** Circumferential size of labral pathology for patients with labral repair included in the study (measurements were performed using clock-face hours)

*Size (clock-face hours)*	*Number of subjects (*n)	*Percentage of subjects (%)*
1	1	5.7
2	82	33.5
3	106	43.3
4	37	15.1
5	4	1.6
6	2	0.8
Total	245	100

Of the 162 labral tears that had 1-year follow-up, 55 (34.0%) were graded as mild, 79 (48.7%) as moderate and 28 (17.3%) as severe. The circumferential size of these labral tears included in this study, as described by the number of ‘hours’ spanned, is presented in Table III. The most common size of tear was a ‘3-hour’ tear, accounting for 77 patients (47.5%) that underwent labral repair.

**Table III.** Circumferential size of labral pathology for patients with labral repair who met a minimum of 1-year follow-up (measurements were performed using clock-face hours)

*Size (clock-face hours)*	*Number of subjects (*n)	*Percentage of subjects (%)*
1	7	4.3
2	49	34.6
3	77	47.5
4	27	16.7
5	1	0.6
6	1	0.6
Total	162	100

### Density of anchor

**Fig. 8.**
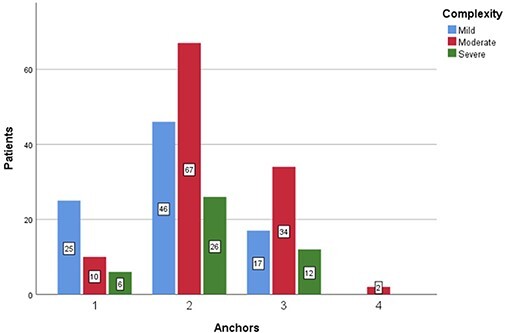
Chart representing the number of anchors used based on the complexity of labral tear: mild (blue), moderate (red) or severe (green).

**Fig. 9.**
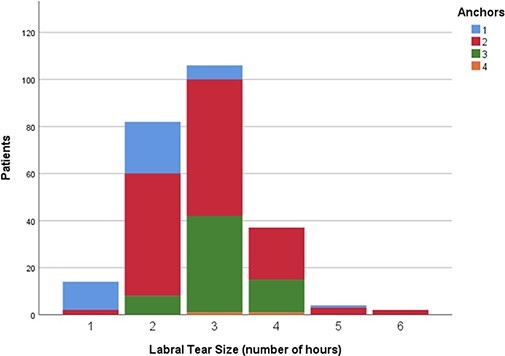
Chart representing the number of anchors used based on the circumferential size of labral tear.

Labral repairs required an average of 2.1 ± 0.67 anchors across the 245 patients included in this study. Forty-one repairs (16.7%) required one anchor, 139 (56.7%) required two anchors, 63 (25.7%) repairs required three anchors, and 2 (0.8%) required four anchors. The number of anchors used based on the complexity and circumferential size of labral tear are presented in Figs 8 and 9, respectively.

**Fig. 10.**
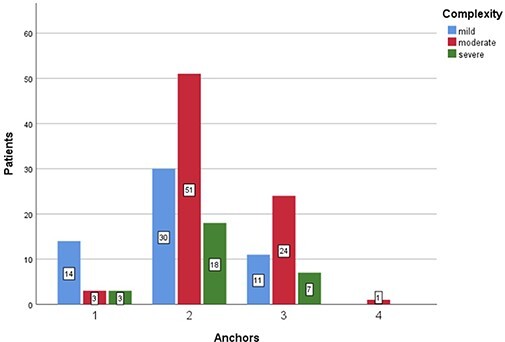
Chart representing the number of anchors used based on the complexity of labral tear.

**Fig. 11.**
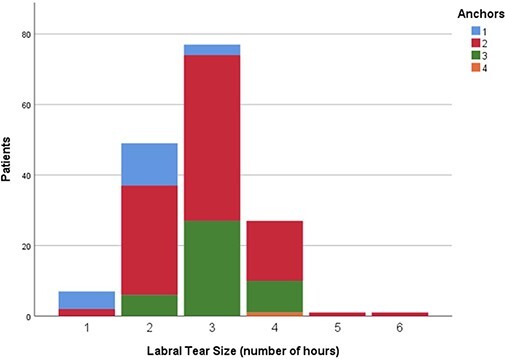
Chart representing the number of anchors used based on the circumferential size of labral tear.

Of the 162 labral tears that had 1-year follow-up, the average labral repair required an average of 2.1 ± 0.62 anchors across the patients included in this study. Twenty repairs (12.3%) required one anchor, 99 (61.1%) required two anchors, 42 (25.9%) repairs required three anchors and 1 (0.6%) required four anchors. The number of anchors used based on the complexity and circumferential size of the labral tear are presented in Figs 10 and 11, respectively.

### Statistical analysis for 1-year follow-up

For the 162 patients evaluated preoperative and at a minimum of 1-year follow-up, significant improvements were reported for all PROs at 1 year (*P* ≤ 0.001), with the results presented in Table IV. This group demonstrated improvements of 24.9 ± 17.2 (*P* ≤ .001) for the HOS-ADL, 33.8 ± 24.1 (*P* ≤ 0.001) for the HOS-Sport, 40.6 ± 27.5 (*P* ≤ 0.001) for the iHOT-12 and −38.2 ± 27.4 (*P* ≤ 0.001) for the VAS. The patients assessed at a minimum of 1 year also reported a satisfaction rate with a surgical intervention of 85.7 ± 23.7.

**Table IV.** Preoperative and postoperative PROs at minimum of 1-year follow-up

	*Preoperative* *(*n* ± SD)*	*Postoperative* *(*n* ± SD)*	*Significance* *(*P*-value)*
HOS-ADL^a^	63.9 ± 14.9	88.7 ± 11.5	≤ .001
HOS-Sport^a^	45.0 ± 19.3	78.8 ± 19.4	≤ .001
iHOT-12^a^	34.5 ± 16.1	75.1 ± 22.3	≤ .001
VAS^a^	57.6 ± 20.5	19.4 ± 19.2	≤ .001

a Graded on a scale of 0–100.

## DISCUSSION

The main findings of the current study demonstrate the safety and efficacy of the ‘mini-Max’ construct for non-absorbable suture anchor allocation during arthroscopic acetabular labral refixation. Primary arthroscopic labral repair with non-absorbable 2.4-mm knotless suture anchors resulted in significantly improved postoperative outcomes (*P* < 0.001). The 162 patients that qualified for a minimum of 1-year follow-up reported statistically and clinically significant improvements for the HOS-ADL, HOS-Sport, iHOT-12, VAS and a satisfaction rate with a surgical intervention of 85.7 ± 23.7.

The postoperative improvement after labral repair in this cohort correlates with other studies reporting on PROs after arthroscopic labral repair procedures [11, 22, 26–28]. Jackson *et*  *al.* reported on 54 patients who underwent arthroscopic primary repair of a torn acetabular labrum using a labral base suture technique with a non-absorbable suture through the base of the labrum and a knotless suture anchor [22]. This study reported that the HOS-ADL significantly improved by 23.2 points, and the HOS-Sport improved by 32.6 points at 2-year follow-up. The VAS pain score had a significant improvement from 6.5 to 2.3 (*P* < 0.0001). Similarly, Rhee *et al*., in a prospective randomized study comparing knot-tying versus knot-less suture anchors for the repair of labrum tears, reported a significant improvement in both groups after 2-year follow-up. The VAS pain score had a significant improvement from 5.9 to 2.3 and 6.4 to 2.9, respectively (*P* < 0.0001). The overall reported HHS for the 37 hips improved 15 points from the preoperative to postoperative evaluation (*P* < 0.001) [11].

A distinct consideration in the cohort studied here is the use of ‘mini-Max’ concepts for the management of the tissues in the repair zone. Pioneering descriptions of labrum repair by Kelly *et al*. using contemporary larger suture anchors (2.9–3.0 mm standard anchors) and tissue penetrators began with the detachment of the labrum from the rim using a blade and re-fixation of the remnant labrum following acetabular rim reduction with a burr [29]. An evolution of the available instrumentation and fixation devices, along with the knowledge gained from following earlier cohorts, informs the modern ‘mini-Max’ technique. This is a technique that maximizes the inherent stability of the tissues and focuses the iatrogenic damage to the zones where healing is required. Although many modern, small implants are available, each time the surgeon drills the acetabular rim, opportunities for error or complications exist, and this cohort demonstrates excellent results with the judicious use of anchors centered within an arc of damage.

A recently performed multicenter cohort study reported that technical limitations in the labrum and bone that are available for anchor placement, suture anchor size and design are factors that may impact the number of suture anchors used in the clinical setting. The authors’ results demonstrated that of the 1636 patients that underwent labral repair, an average of 2.7 ± 0.9 anchors across all surgical centers were used. Suture anchors accounted for 76% of all cases included in the study while all-suture fixations were performed in 22% of the cases and 2% received bio-composite anchors. Six of the seven participating surgeons used predominately suture anchors for labral refixation, which demonstrated a lower number of anchors used with 2.6 ± 0.82 anchors per repair versus 3.3 ± 0.94 sutures per repair for the all-suture technique [20]. While it is widely accepted that larger labral tears require a higher number of fixation anchors, the ‘mini-Max’ technique enables utilization of the fewest essential suture anchors to minimize disruption of the capsulolabral tissue while ensuring stable tissue approximation and healing [20].

During the last decade, arthroscopic procedures for acetabular labrum repair have demonstrated improved clinical outcomes and joint space preservation at short-term follow-up [22, 26–28]. Restoring the labrum as close to its original anatomy is essential in order to preserve and reestablish the labrum’s function. Anchor placement and suture management play a crucial role in restoring the labrum’s suction seal [7, 20]. Advances in surgical techniques for hip arthroscopy, as well as recently introduced suture anchors and surgical devices, have contributed with these promising results [7, 11, 30]. However, a specific technique for the most optimal repair remains to be defined. The ‘mini-Max’ technique utilizing non-absorbable knotless suture anchors for hip arthroscopy allows restoration of labrum’s functional suction seal with a minimal invasive technique and improved PROs at 1-year follow-up.

There are limitations that need to be considered when interpreting the results. The current study was a retrospective review of PROs that introduced several potential limitations that included loss to follow-up and selection bias. The mean follow-up time for this study was 1 year. However, current literature reporting on 1- and 2-year follow-ups have shown comparable results in between these two time frames [11, 22, 31]. Also, associated intraoperative procedures, which required concomitant surgical corrections, made difficult the evaluation of isolated effects of arthroscopic labral repair. The lack of a comparison group adds a limitation; however, this was not the purpose of this study.

## CONCLUSIONS

This study adds to the current literature by demonstrating clinical outcomes obtained after primary arthroscopic acetabular labrum repair using a modern ‘mini-Max’ technique after 1-year follow-up. At the same time, this study provides evidence regarding the ability to obtain an adequate fixation with preservation of the chondral–labrum junction and a functional suction seal when utilizing non-absorbable knotless suture anchors for hip arthroscopy.

## Data Availability

The data underlying this article are available in the article and in its online supplementary material.

## APPENDIX A

### Post-Operative Care

Patients were seen in office by the senior author (RPM) the day after surgery, followed by initiation of physical therapy that same day. All hip arthroscopy patients were prone in bed for the first postoperative night with abduction cylinder and boots. Patients were limited to 20 lbs. Foot-flat weight bearing with crutches for the first 2-weeks following surgery. All patients were pre-fit for a hip orthosis (T-Scope Hip; Breg, Inc) by a trained medical equipment professional, began wearing the brace immediately following surgery and were checked for fit during the initial postoperative visit. The brace was set to allow full hip extension and 90 degrees of hip flexion. The patients were instructed to wear the brace at all times outside of continuous passive motion or in formal physical therapy sessions. Extended use of the crutches and hip brace for an additional 4-weeks was initiated at the senior authors discretion for patients undergoing microfracture and/or labral reconstruction.

For all patients, passive motion was initiated immediately after surgery for prevention of adhesion formation within the joint. A continuous passive motion machine (CPM) was used for the first 2 weeks for six hours-a-day (3 - 2-hour sessions) following hip arthroscopy. Passive hip pendulums for 1 hour-a-day (3–20 minutes sessions) were performed based on a previously performed study.^30^ Along with passive motion, patients were instructed to lay prone 1 hour-a-day (3–20 minutes sessions). Formalized physical therapy was for 1 visit weekly for the first 6 weeks, increasing to 2 visits weekly for up to 4 months. Physical therapy focused on the patient’s native gait pattern, the needs of their functional positioning during work, life and sport, and any observable or modifiable conflicts. Core strengthening, lumbopelvic control, and functional ROM and performance were key tenants in the rehabilitation protocol.

Postoperative medications are prescribed and filled prior to surgery for hip arthroscopy patients. The general regimen includes a pain medication, a nausea medication, and an NSAID as a prophylaxis for heterotopic ossification of soft tissues of the hip after surgery. Patients took all medications as prescribed on an as needed basis except the NSAID pill which is taken as prescribed on a scheduled dose with food.
